# Melatonin Promotes the Therapeutic Effect of Mesenchymal Stem Cells on Type 2 Diabetes Mellitus by Regulating TGF-β Pathway

**DOI:** 10.3389/fcell.2021.722365

**Published:** 2021-10-15

**Authors:** Balun Li, Xuedi Cheng, Aili Aierken, Jiaxin Du, Wenlai He, Mengfei Zhang, Ning Tan, Zheng Kou, Sha Peng, Wenwen Jia, Haiyang Tang, Jinlian Hua

**Affiliations:** ^1^Shaanxi Centre of Stem Cells Engineering and Technology, College of Veterinary Medicine, Northwest A&F University, Xianyang, China; ^2^Department of Animal Engineering, Yangling Vocational and Technical College, Xianyang, China; ^3^Department of Veterinary Medicine, College of Animal Sciences, Institute of Preventive Veterinary Sciences, Zhejiang University, Hangzhou, China; ^4^Shanghai East Hospital, East Hospital Affiliated to Tongji University, Shanghai, China; ^5^State Key Laboratory of Respiratory Disease, Guangzhou Institute of Respiratory Health, The First Affiliated Hospital of Guangzhou Medical University, Guangzhou, China

**Keywords:** melatonin, adipose-derived mesenchymal stem cells, type 2 diabetes mellitus, TGF-β, inflammation, canine

## Abstract

Abundant evidence proves the therapeutic effect of adipose-derived mesenchymal stem cells (ADMSCs) in the treatment of diabetes mellitus. However, the problems have not been solved that viability of ADMSCs were inconsistent and the cells quickly undergo senescence after *in vitro* cell culture. In addition, the therapeutic effect of ADMSCs is still not satisfactory. In this study, melatonin (MLT) was added to canine ADMSC culture medium, and the treated cells were used to treat type 2 diabetes mellitus (T2DM). Our research reveals that adding MLT to ADMSC culture medium can promote the viability of ADMSCs. This effect depends on the binding of MLT and MLT receptors, which activates the transforming growth factor β (TGF-β) pathway and then changes the cell cycle of ADMSCs and improves the viability of ADMSCs. Since ADMSCs were found to be used to treat T2DM by anti-inflammatory and anti-endoplasmic reticulum (ER) stress capabilities, our data demonstrate that MLT augment several effects of ADMSCs in remission hyperglycemia, insulin resistance, and liver glycogen metabolism in T2DM patients. This suggest that ADMSCs and MLT-ADMSCs is safe and vabulable for pet clinic.

## Introduction

The pathogenesis of type 2 diabetes mellitus (T2DM) includes glucose metabolism disorder, oxidative stress, endoplasmic reticulum (ER) stress, and inflammation (Hu et al., 2018; Elshemy et al., 2021). Therefore, the interaction among multiple mechanisms leads to liver glucose metabolism disorder, insulin resistance, damage to the function of islet cells and hyperinsulinism. Islet β cells are the only cells that produce insulin in the body, and their functional damage directly leads to T2DM. Notably, the levels of ER and inflammation are critical for maintaining β-cell survival, and islet β-cell damage caused by ER damage and chronic inflammation has become an important factor in T2DM (Oyadomari et al., 2002). The ER is an organelle in eukaryotic cells and is involved in protein synthesis, modification, processing, and quality control (Saito and Imaizumi, 2018). ER stress at the normal level can promote cell self-renewal, while long-term or severe ER stress can cause cell dysfunction and death (Li et al., 2020). Inflammation promotes the body to activate adaptive immunity and repair damaged tissues. However, excessive inflammation and chronic inflammation are also the causes of many chronic diseases, including chronic inflammatory rheumatism, diabetes mellitus (DM), and neurodegenerative diseases. ER stress is usually associated with inflammation. Both ER stress and inflammation represent short-term adjustments to body imbalance and are harmful when they are persistent or chronic (Chovatiya and Medzhitov, 2014). Studies have shown that inflammation occurs during ER stress and causes body damage, but the mechanism is still unclear (Cai et al., 2016; Cao et al., 2016; Wei et al., 2018).

Mesenchymal stem cells (MSCs) are pluripotent stem cells belonging to the mesoderm. MSCs have the potential to differentiate into bone cells, chondrocytes, adipocytes, muscle cells and other cells (Strem et al., 2005). MSC transplantation has been proven feasible for the treatment of severe traumatic diseases, autoimmune diseases, DM, and neurodegenerative diseases (Xie et al., 2017; Yang et al., 2021). There are many types of MSCs. Currently, most studies focus on umbilical cord blood mesenchymal stem cells (UCBMSCs), bone marrow mesenchymal stem cells (BMSCs), adipose-derived mesenchymal stem cells (ADMSCs), dental pulp MSCs, and limbal MSCs. Compared with MSCs from other sources, ADMSCs are obtained by liposuction and have the advantages of wide sources, convenient acquisition, low immunogenicity, and low ethical controversy.

Studies have shown that BMSC therapy improved insulin secretion, activate the insulin signaling pathway and enhance glucose transport, thereby reversing hyperglycemia in T2DM rats (Si et al., 2012; Hao et al., 2013). Fat derived MSCs improve hyperglycemia by regulating hepatic glucose metabolism in T2DM rats. Cell-free therapy (MSC-CM) based on biologically active factors secreted by stem cells and progenitor cells restore hyperglycemia and improve oxidative stress in T2DM rats (Elshemy et al., 2021). In addition, studies have revealed that autologous UCBMSC therapy can reduce patients’ insulin dosage (Guan et al., 2015). However, the current MSC transplantation treatment still has some problems, including an uneven quality of MSCs used for treatment, an incomplete quality evaluation system for MSCs, and unclear treatment mechanism (Zakrzewski et al., 2019). For example, studies have shown that MSCs derived from T2DM patients contain more oxysterols, affecting the differentiation ability of MSCs (Luchetti et al., 2009; Murdolo et al., 2013). The source and quality of cells become major factors limiting the use of MSCs in clinical treatment. There is an urgent need for further explorations of the mechanism of MSC therapy to provide strong evidence for the application of MSCs in clinical medicine.

Melatonin (MLT) is an endogenous indolamine synthesized by tryptophan that is secreted by the pineal gland into blood circulation and regulates many physiological functions (Luchetti et al., 2010; Kumar Jha et al., 2015). Regarding its physiological role, MLT is a key regulator of the circadian rhythm (Reiter et al., 2010). For example, MLT plays a protective role in the body, including protecting the kidneys, through its powerful anti-inflammatory and antioxidative stress capabilities (Reiter et al., 1994, 2010; Sener et al., 2002; Luchetti et al., 2010; Galano et al., 2011; Mauriz et al., 2013). In addition, numerous studies have confirmed that MLT affects the occurrence and development of cells and the biological functions of MSCs and oocytes in various ways (Luchetti et al., 2009, 2014; Shi et al., 2009; Rocha et al., 2013; Tian et al., 2014; Maria et al., 2018; Liu et al., 2019). Studies have found that MLT acts as a mitochondrial antioxidant through the ERK-MAPK signaling pathway preventing apoptosis (Luchetti et al., 2009). MLT promotes the osteogenic differentiation of MSCs and protects BMSCs from bone injury (Knani et al., 2019). In addition, studies have shown that MLT predominantly regulates the differentiation and survival of MSCs through the Wnt/β-catenin pathway, MAPKs and TGF-β signaling (Luchetti et al., 2014). Our previous studies show that MLT prevents canine-derived ADMSCs from aging by activating NRF2 and inhibiting ER stress, restores the bone differentiation ability of aging ADMSCs, and promotes the effect of MSC therapy (Fang et al., 2018). MLT plays an important role in the *in vitro* culture of MSCs. However, the mechanism by which MLT affects the viability of MSCs remains unclear. Transforming growth factor β (TGF-β) family promotes cell proliferation, differentiation, migration and survival by controlling the expression and activity of key transcription factors in the TGF-β pathway (Blobe et al., 2000; Li et al., 2006). The upstream TGF-β pathway comprises the TGF-β ligand, type 1 TGF-β receptor (TGF-βR1) and type 2 TGF-β receptor (TGF-βR2), and the downstream TGF-β pathway comprises Smad and Smad-related transcription factors, which participate in cell proliferation and differentiation through transcriptional regulation (Budi et al., 2017; Derynck and Budi, 2019). Studies have shown that TGF-β plays a key role in the proliferation of MSCs, and the inhibition of TGF-β receptors slows the proliferation of MSCs (Ng et al., 2008; Luchetti et al., 2014).

In this study, canine-derived ADMSCs were cultured in a medium containing MLT *in vitro*, and the results showed that MLT promoted the viability of the ADMSCs by regulating the TGF-β pathway. This study shows that MLT promotes ADMSCs to treat T2DM by restoring islet and liver ER stress and inflammation. This study provides reliable and complete evidence supporting the use of MLT pretreatment as a part of MSC therapy. In addition, dogs are used as a DM model, and the usage of canine-derived ADMSCs can indirectly reflect the effect of the allogeneic transplantation of ADMSCs. Our research provides new evidence for clinical MSC therapy.

## Materials and Methods

### Experimental Animals

Sixty-five 8-week-old Kun-Ming (KM) male mice (25 ± 2 g) were purchased from Chengdu Dossy Experimental Animal Co., Ltd. Eighteen 1-year-old male hybrid dogs weighing 5.0 ± 0.5 kg, provided by the Experimental Animal Center of Northwest A&F University, were used to establish canine DM models and perform safety tests. Two 6-month-old female hybrid dogs weighing 3.0 ± 0.5 kg, provided by the Experimental Animal Center of Northwest A&F University, were used for the ADMSC separation. All animal experimental protocols were performed in strict accordance with the Guide for the Care and Use of Laboratory Animals (Ministry of Science and Technology of the People’s Republic of China, Policy No. 2006 398). All animals were maintained in a conventional sanitary facility with the required consistent temperature and relative humidity. All animal experimental protocols were reviewed and approved by the Ethics Committee (no. 2015-mkrm01) of Northwest A&F University for the Use of Laboratory Animals. This experiment followed the international guidelines for animal studies (National Research Council (US) Institute for Laboratory Animal Research, 2004).

### Cell Separation and Culture

Adipose-derived mesenchymal stem cells were derived from the abdominal subcutaneous adipose tissue of two 6-month-old female hybrid dogs. The detailed ADMSC separation steps and ADMSC identification were described in our previous report (Wei et al., 2016). The cells were cultured in α-MEM (Invitrogen, Carlsbad, CA, United States) complete medium at 37°C in a 5% CO_2_ incubator (Peng et al., 2012; Fang et al., 2018). When the cells were attached to the bottom of the plate at approximately 80%, a 1:3 passage was performed. We treated and used the fourth-passage cells. MLT was added to the culture medium 72 h before transplantation, sample collection, and staining.

### Melatonin Treatment of Adipose-Derived Mesenchymal Stem Cells

At the P4 passage, 1 × 10^5^ cells were inoculated into a 48-well plate. ADMSCs were treated with 1 μM MLT, and the MLT-containing medium was replaced every 24 h for three times. After 72 h of MLT treatment, bright field images of cells were taken and Giemsa staining and ethynyldeoxyuridine (EdU) staining were performed. We used 60 mm cell dishes to culture the ADMSCs and collect the total cell RNA to complete the subsequent experiments (Fang et al., 2018).

### Ethynyldeoxyuridine Staining

According to the instructions provided by the reagent supplier, (RiboBio, Guangzhou, China) we used logarithmic growth phase cells; we inoculated 0.5 × 10^4^ cells into each well of a 96-well plate and cultured the cells to a density of 60–70% (Peng et al., 2012). EdU solution (1000:1) was diluted with serum-containing α-MEM medium and added to a 96-well plate. Then, the cells were incubated for 2 h, and the culture medium was discarded. The cells were washed with phosphate-buffered saline (PBS) (washed twice for 5 min per wash), cell fixation solution (4% paraformaldehyde in PBS) was added, and the samples were incubated at room temperature. Thirty minutes later, 2 mg/mL glycine were added to the cells, and the samples were incubated on a decolorizing shaker for 5 min, glycine was discarded, and the cells were washed with PBS for 5 min. Apollo staining solution was added to the cells, the samples were incubated for 30 min in the dark at room temperature, and then, the staining solution was discarded. Then, the cells were added to a 0.5% Triton X-100 decolorizing shaker and washed three times for 10 min per wash, and the permeate was discarded. Hoechst 33342 was added to the cells, and the samples were incubated for 30 min in the dark at room temperature. The staining solution was discarded, and the cells were washed once with PBS.

### Cell Growth Curve

Adipose-derived mesenchymal stem cells were cultured in 24-well plates at a density of 0.5 × 10^4^ cells per well. A cell growth curve was used to investigate the proliferation ability every 24 h. The ADMSCs were trypsinized every day, and the total number of cells was determined for seven consecutive days (Wei et al., 2016; Wu et al., 2021).

### Giemsa Stain

According to the instructions provided by the reagent supplier (ZHONGHUIHECAI, China), we used logarithmic growth phase cells, inoculated 0.5 × 10^4^ cells into each well of a 96-well plate and cultured the cells to a density of 60–70%. The Giemsa mother solution was diluted ten times with PBS to obtain the Giemsa working solution. We used 100 μL of the Giemsa working solution to fix the cells. After 1 min, 100 μL PBS were added to the cell culture dish. After 30 min, the staining solution was discarded, the cells were washed twice with PBS, and the cell status was observed under a microscope.

### Quantitative Real-Time Polymerase Chain Reaction Analysis

According to the manufacturer’s instructions, the total RNA was extracted from the ADMSCs by TRIzol reagent (Takara, Japan), and a reverse transcriptase reagent kit (Thermo Fisher Scientific) was used. Quantitative real-time polymerase chain reaction (qRT-PCR) was carried out using a CFX96 Real-Time polymerase chain reaction (PCR) system as follows: predenaturation at 94°C for 5 min, followed by 39 cycles for 30 s at 94°C, annealing for 30 s at 58°C and 30 s at 70°C for extension. β-Actin was used as an internal control. The comparative CT values from the qRT-PCR were used to measure the relative gene expression (Wei et al., 2016; Zhu et al., 2021). The primers are listed in Supplementary Table 1.

### Type 2 Diabetes Mellitus Animal Model

Twelve 1-year-old male hybrid dogs and 65 KM male mice were used in the T2DM animal model. All animals were placed in the Animal Experiment Center of Northwest A&F University at constant temperature (25 ± 2°C) and constant photoperiod (12:12 h light-dark cycle) and were given adequate drinking water. To eliminate external factors, the dogs were bred adaptively before the experiment.

Twelve 1-year-old male hybrid dogs and 65 KM male mice were divided into the following five groups: (1) normal control, (2) T2DM, (3) ADMSCs, (4) MLT-ADMSCs, and (5) SB-MLT-ADMSCs. The first four groups included three dogs and 15 mice, and the fifth group included five mice. To induce the T2DM model, the last four groups were fed a high fat diet for 8 weeks combined with intravenous transplantation of streptozotocin (STZ). The transplant amount per dog was 25 mg/kg/day for 2 days, and that per mouse was 35 mg/kg/day for 2 days (Sun et al., 2018). The STZ dose for the dogs was obtained by using different doses of STZ transplantation (Supplementary Figure 1). STZ was diluted with sodium citrate buffer, and the dogs and mice were fasted for 24 h before injection. After 1 week of modeling, the modeling effect was identified. In the latter three groups, ADMSCs (dogs: transplant 1 × 10^7^ cells suspended in 10 mL sterile 0.9% NaCl, mice: transplant 2 × 10^6^ cells suspended in 0.2 mL sterile 0.9% NaCl) were injected through the brachial vein of the dogs’ forearm and tail vein in the mice. During the model preparation and treatment, we continuously monitored the changes in body weight and water and food intake.

### Safety Test

Six 1-year-old male hybrid dogs were divided into three groups: (1) Normal Control, (2) ADMSCs, (3) MLT-ADMSCs. All dogs were bred adaptively for 1 week. In the latter two groups, ADMSCs (ADMSCs: transplanted 1 × 107 ADMSCs suspended in 10 mL sterile 0.9% NaCl, MLT-ADMSCs: transplanted 1 × 107 MLT-ADMSCs suspended in 10 mL sterile 0.9% NaCl) passed through the dog’s forearm Brachial vein injection. To determine trace the ADMSCs transplanted into the body, the ADMSCs were digested with 0.25% trypsin and resuspended before transplantation. Then the PKH26 red fluorescent cell linker kit was used to label the ADMSCs (Sigma-Aldrich, United States) before transplantation. On the 0th day, 30th day and 60th day of cell transplantation, the blood of each group of dogs was collected for blood routine examination (Prokan, China) and blood biochemical test (Mindray, China). After the 60th day of cell transplantation, all dogs were euthanized (intravenous overdose of KCl). Collect dog liver, spleen, kidney, and pancreas tissues for frozen section. Subsequently, sections were stained with Hoechst 33342 at 0.5 μg/mL before being observed under a microscope.

### Histological Analysis

The liver and pancreas tissues were fixed in 4% paraformaldehyde, gradually dehydrated, embedded in paraffin, cut into 4 μm sections, and subjected to hematoxylin/eosin (H&E) staining. Periodic acid Schiff (PAS) staining was performed according to the manufacturer’s protocols for liver sections (Solarbio, China) (Fang et al., 2018; Yan et al., 2019).

For the cellular immunofluorescence, the cells were fixed in 4% paraformaldehyde in phosphate-buffered saline (PBS) at room temperature (RT) for 10 min, washed three times with PBS, and then permeabilized for 15 min with 0.1% Triton-X 100 (Sigma-Aldrich, St. Louis, MO, United States) in PBS at RT. The cells were blocked with PBS supplemented with 4% bovine serum albumin for 30 min and incubated with primary antibodies against MT1 (1:100, Boster, China) and MT2 (1:100, Boster, China) at 4°C for 16 h. After washing with PBS three times, the cells were incubated with secondary antibodies for 1 h at 37°C in the dark. Following another three washing steps with PBS, nuclear counterstaining was performed with 1 μg/mL Hoechst 33342 (Sigma-Aldrich). The fluorescence images were obtained by Evos f1 fluorescence microscopy (AMG, United States).

For the tissue immunofluorescence, liver and pancreas sections were subjected to baking, dewaxing, and repair of antigens with sodium citrate buffer (0.01 M, pH 6.0). The liver sections were incubated with a rabbit anti-rat glucose transporter 4 (GLUT4) antibody, and the pancreas sections were incubated with a rabbit anti-rat insulin antibody (1:100, Proteintech, China) at 4°C overnight. Then, the sections were washed and incubated with Alexa Fluor 555-conjugated donkey anti-rabbit IgG or FITC-conjugated goat anti-rabbit IgG (Invitrogen) for 1 h. Subsequently, the sections were stained with Hoechst 33342 at 0.5 μg/mL before being observed under a microscope.

For the immunohistochemistry, liver and pancreas sections were subjected to baking, dewaxing, and repair of antigens with sodium citrate buffer (0.01 M, pH 6.0). The tissue was blocked with animal serum after eliminating the effects of endogenous peroxidase with 3% H_2_O_2_. The liver and pancreas sections were incubated with rabbit anti-rat interleukin-10 (IL-10) (1:200, Proteintech, China), tumor necrosis factor-α (TNF-α), interleukin-6 (IL-6) (1:200, CST, United States), glucose-regulated protein 78 (GRP78), C/EBP-homologous protein (CHOP), and activating transcription factor 6 (ATF-6) (1:200, Bioss, China) antibodies at 4°C overnight. Then, the cells were incubated with horseradish peroxidase-labeled streptavidin working solution after washing three times with PBS. The detection was performed using 3′-diaminobenzidine (ZLI-9018; Beijing Zhongshan Golden Bridge Biotechnology Co., Ltd.), and the nucleus was stained with hematoxylin. Finally, the samples were dehydrated and covered. The tissues were analyzed under a light microscope (Nikon, Japan) (Wei et al., 2021).

### Statistical Analysis

When the main effects were significant, a one-way analysis of variance (ANOVA) was used, followed by Newman–Keuls multiple range tests. A Student’s *t*-test was used when comparing the means of two groups. All data are presented as the mean ± SE, and statistical significance is shown as follows: ns > 0.05; ^∗^*p* < 0.05; ^∗∗^*p* < 0.01; and ^∗∗∗^*p* < 0.001. All data were analyzed by GraphPad Prism software (La Jolla, CA, United States) and represent a minimum of three different experiments.

## Results

### Melatonin Treatment Can Enhance the Viability of Adipose-Derived Mesenchymal Stem Cells Cultured *in vitro*

**FIGURE 1:**
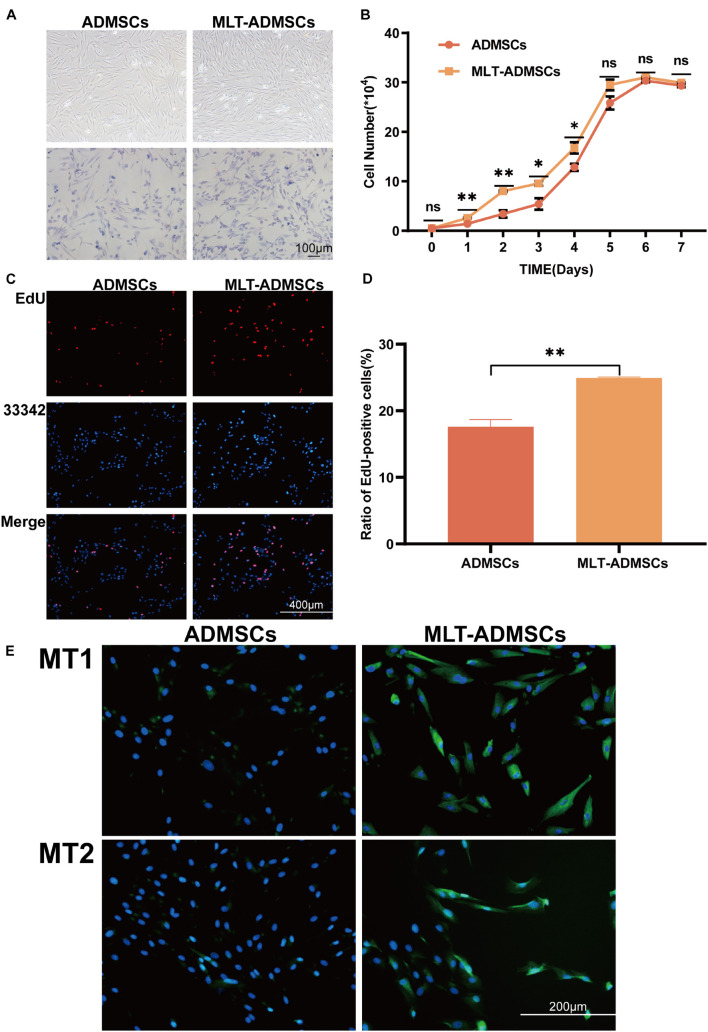
MLT treatment can enhance the viability of ADMSCs cultured *in vitro*. **(A)** Cell morphology (ADMSCs and MT-ADMSCs). **(B)** Cell growth curve. **(C)** EdU staining. **(D)** Quantitative analysis of EdU. **(E)** MT1/MT2 immunofluorescence staining. Values in this figure are the mean ± SE; *n* = 3 per group; ns > 0.05, ^∗^*p* < 0.05, ^∗∗^*p* < 0.01 determined by a repeated-measures ANOVA.

Compared with the normal cultured ADMSCs, the MLT treatment did not change the morphology but increased the number of the ADMSCs (Figure 1A). The ADMSCs maintained rapid proliferation. and MLT treated ADMSCs proliferated faster than the control group in the first 5 days and particularly be observed in the first 4 days (Figure 1B). As shown in the results of the EdU staining, the cell proliferation rate of the MLT treatment group was faster than that in the normal ADMSC group (Figures 1C,D). The main MLT receptors include MLT receptor 1A (MT1) and MLT receptor 1B (MT2). By staining MT1/MT2 with immunofluorescence, we found that MLT bound MLT-ADMSCs effectively in the MLT treatment group (Figure 1E). The results show that MLT binds the MT1/MT2 receptors of ADMSCs and promotes the viability of ADMSCs cultured *in vitro*.

### Melatonin Enhances the Recovery Effect of Adipose-Derived Mesenchymal Stem Cells on Clinical Symptoms and Hyperglycemia in Type 2 Diabetes Mellitus Mice and Promotes Islet Reconstruction

**FIGURE 2:**
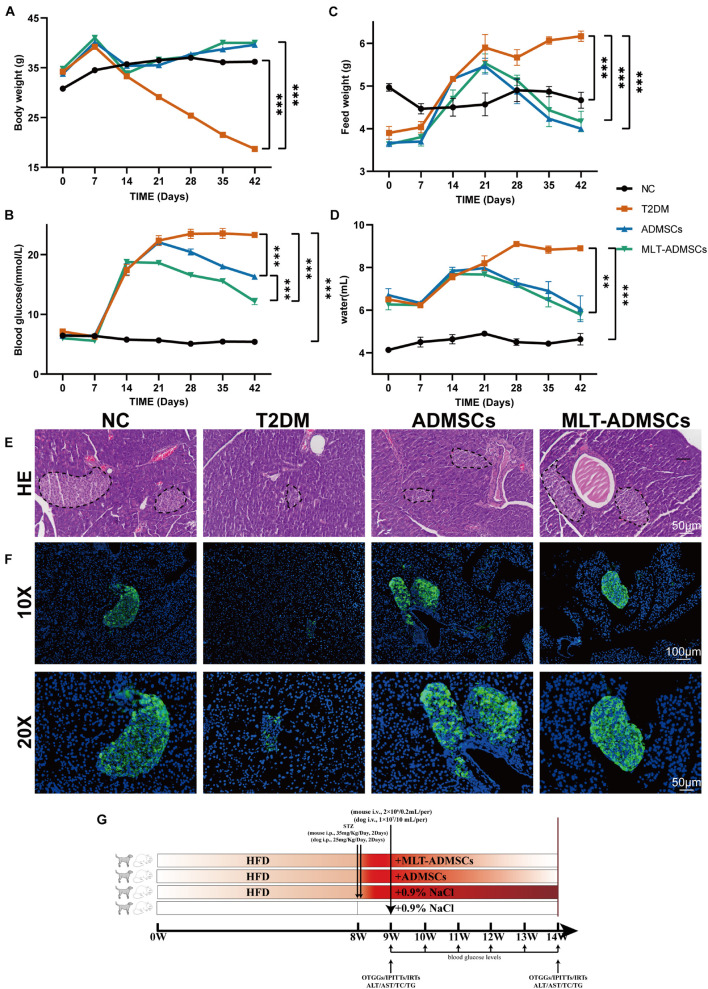
MLT enhances the recovery effect of ADMSCs on clinical symptoms and hyperglycemia in T2DM mice and promotes islet reconstruction. **(A)** Continuous monitoring of the weight level of mice. **(C)** Changes in the feed intake of mice. **(D)** Changes in the water intake of mice. **(B)** Random blood glucose changes in mice. **(E)** Pancreas H&E staining. **(F)** Pancreas immunofluorescence insulin. **(G)** Experimental design. Values in this figure are the mean ± SE; *n* = 10 mice per group; ^∗∗^*p* < 0.01, ^∗∗∗^*p* < 0.001 determined by a repeated-measures ANOVA.

To determine the therapeutic effect of ADMSCs on T2DM, T2DM model mice were prepared by a high-fat diet (HFD) combined with low-dose STZ injection. The data show that we successfully established a T2DM mouse model (Figures 2A–F). The weight of the mice reached approximately 40 g after 8 weeks of the HFD and was significantly reduced after the STZ injection (Figure 2G). In addition, the diet of the mice increased, and the blood sugar level exceeded 16.7 mmol/L, which is consistent with the typical symptoms of T2DM (Figures 2A–D). The ADMSCs and MLT-ADMSCs effectively reduced the weight of the mice (Figure 2A). Similarly, the increase in the diet of the mice caused by T2DM was alleviated in the ADMSC group and MLT-ADMSC group (Figures 2B,C). The occurrence of T2DM is mainly assumed in the presence of hyperglycemia and insulin resistance. The data show that treatment with ADMSCs alleviates hyperglycemia in mice, which was manifested by inhibiting the further deterioration of the disease and maintaining the blood sugar level at the initial level of T2DM. Surprisingly, ADMSCs treated with MLT had a stronger hypoglycemic effect on T2DM. After treatment with MLT-ADMSC, the hyperglycemia of the mice was significantly improved (Figure 2D). The hyperglycemia that occurs in T2DM is mainly caused by islet damage and insulin resistance in peripheral tissues. In this study, H&E staining and immunofluorescence staining were used to detect pancreatic islet damage and insulin secretion in mice. The results showed that after the HFD combined with the STZ injection, the pancreatic islets were damaged in the mice, and the amount of insulin secretion was significantly reduced. The results showed that after T2DM was treated with PBS sham operation, blood sugar continued to rise, pancreatic islets decreased, β cells were lost, and insulin secretion was significantly reduced. After treatment with ADMSC treatments, the mice’s pancreatic islet injury was significantly recovered, the island structure was clear, the number of β cells was restored, and the amount of insulin secretion was significantly restored. In addition, we found that insulin secretion after the MLT-ADMSC treatment was significantly restored and basically returned to the normal levels (Figures 2E,F). Various data show that the ADMSC treatment restored the typical symptoms of T2DM in mice, including restoring their diet, improving their weight, lowering the blood sugar levels, rebuilding islets, and restoring insulin secretion.

### Melatonin Enhances the Recovery Effect of Adipose-Derived Mesenchymal Stem Cells on Insulin Resistance in Type 2 Diabetes Mellitus Mice

**FIGURE 3:**
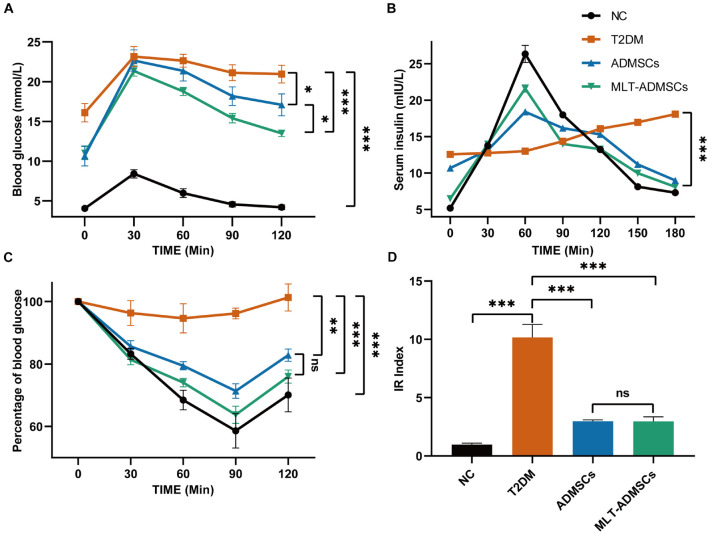
MLT enhances the recovery effect of ADMSCs on insulin resistance in T2DM mice. **(A)** OTGG. **(B)** IRTs. **(C)** IPITT. **(D)** IR index in each group, HOMA-IR index = [FBG (in mmol/L) × FINS (in units/L)]/22.5. The blood glucose level in each group was detected after fasting for 3 h. Values in this figure are the mean ± SE; *n* = 10 mice per group; ns > 0.05, ^∗^*p* < 0.05, ^∗∗^*p* < 0.01, ^∗∗∗^*p* < 0.001 determined by a repeated-measures ANOVA.

When T2DM occurs, the body develops insulin resistance, and the utilization rate of insulin is reduced, resulting in an increase in blood sugar that cannot be reduced. In addition, insulin resistance causes islet β cell fatigue and further damages islet β cells. The relative lack of insulin changes to an absolute lack of insulin and aggravates T2DM. Related indicators of insulin resistance, including the OTGG, IRT, IPITT, and HOME-IR index, were used to assess the therapeutic effect of ADMSCs. In addition, the differences in the therapeutic effects of ADMSCs and MLT-ADMSCs were compared. After the treatment with ADMSCs, insulin resistance in the T2DM mice was reduced. Interestingly, compared with the ADMSCs, the MLT-ADMSCs had a stronger recovery effect on insulin resistance (Figures 3A,C). When the body produces insulin resistance, its sensitivity to insulin decreases. ADMSCs treatment restored the secretion and utilization of insulin. MLT enhances the effect of ADMSCs (Figure 3B). The HOME-IR index results showed that the ADMSCs restored the insulin sensitivity of the mice, and the MLT-ADMSC group exhibited a stronger effect (Figure 3D). These various data indicate that ADMSCs can promote the recovery of insulin resistance and significantly restore insulin sensitivity in mice. Surprisingly, the MLT treatment significantly enhanced this role of ADMSCs.

### Melatonin Enhanced the Recovery Effect of Adipose-Derived Mesenchymal Stem Cells on Glucose Metabolism in Type 2 Diabetes Mellitus Mice and Repaired Liver Damage and Lipid Metabolism Disorder Caused by Type 2 Diabetes Mellitus

**FIGURE 4:**
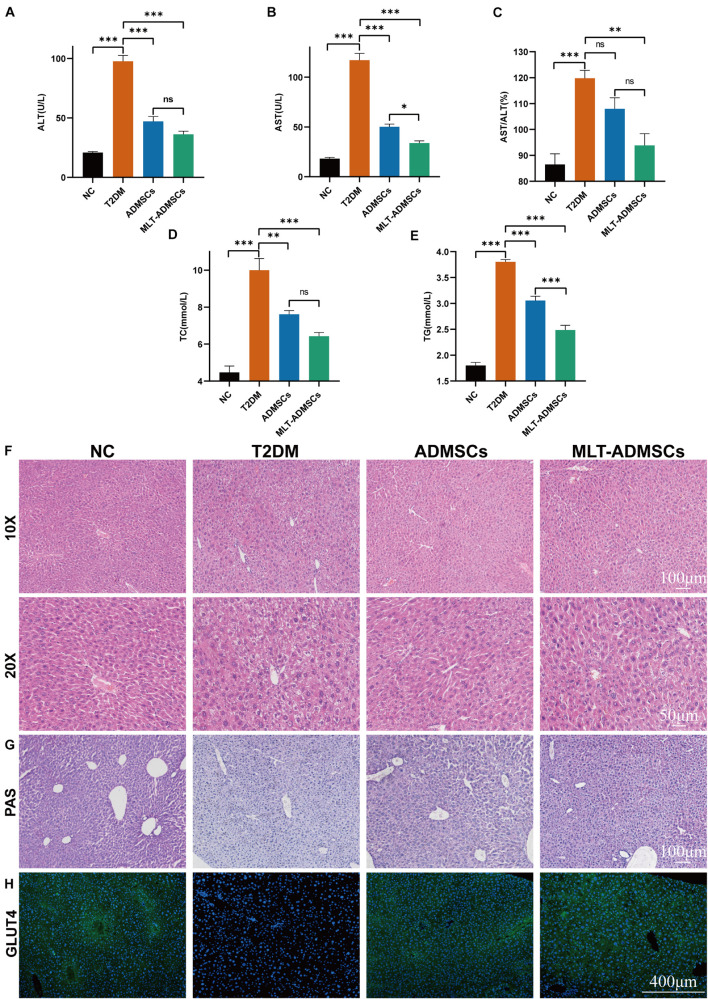
MLT enhanced the recovery effect of ADMSCs on glucose metabolism in T2DM mice and repaired the liver damage and lipid metabolism disorders caused by T2DM. **(A)** Serum ALT. **(B)** Serum AST. **(C)** AST/ALT. **(D)** Serum TC. **(E)** Serum TG. **(F)** Liver H&E staining. **(G)** Liver PAS staining. **(H)** Liver immunofluorescence GLUT4. Values in this figure are the mean ± SE; *n* = 10 mice per group; ns > 0.05, ^∗^*p* < 0.05, ^∗∗^*p* < 0.01, ^∗∗∗^*p* < 0.001 determined by a repeated-measures ANOVA.

The liver is an important organ of glucose metabolism. The liver plays an important role in maintaining blood sugar balance by regulating the absorption, storage, production and metabolism of glucose. When T2DM occurs, the liver is damaged, which leads to impaired glucose metabolism. The results revealed that when T2DM occurred, the liver was damaged, and the biochemical indicators changed, manifesting as increases in ALT and AST (Figures 4A–C). ADMSCs treatment restored the liver function of mice, and MLT-ADMSCs had better effect (Figures 4A–C). In addition, when T2DM occurs, the liver is damaged, the liver cells are fused necrotic, swollen, nucleus loose, and balloon-like degeneration. ADMSCs treatment promote liver damage repair and liver cell regeneration, MLT enhances this effect (Figure 4F). Our previous studies have shown that ADMSCs restored liver damage caused by carbon tetrachloride (Yan et al., 2019). In this study, the ADMSC treatment alleviated the liver damage caused by T2DM, proving the extensive therapeutic effects of ADMSCs on the liver. When T2DM occurs, with the decreasing ratio of insulin to glucagon, lipolysis accelerates, and numerous fatty acids and glycerol enter the liver. Too many fatty acids esterified into triglycerides causes hyperlipidemia and easily leads to complications of T2DM, such as atherosclerosis. The ADMSC treatment promoted the recovery of the serum TC and TG levels, and MLT enhanced this effect (Figures 4D,E). Previous studies have shown that ADMSCs promoted the body’s sensitivity to insulin and improve the body’s insulin resistance (Figure 3), MLT promote the effect of ADMSCs. To study how ADMSCs improve insulin resistance and insulin sensitivity, hepatic glycogen accumulation and GLUT4 expression were detected, and the ADMSC treatment restored the glycogen synthesis disorder and GLUT4 expression caused by T2DM (Figures 4G,H). These results indicate that ADMSCs repaired liver damage caused by T2DM and restored the glucose metabolism and lipid metabolism functions of the liver. The repair of liver leads to the recovery of glucose and lipid metabolism, thereby alleviating insulin resistance, restoring insulin sensitivity, promoting islet regeneration, and restoring insulin secretion. In summary, ADMSCs treat T2DM by restoring liver function and rebuilding islets. Moreover, MLT-ADMSCs exhibit a stronger therapeutic effect.

### Melatonin Promotes the Therapeutic Effect of Adipose-Derived Mesenchymal Stem Cells by Enhancing the Anti-inflammatory and Anti-endoplasmic Reticulum Stress Abilities of Islets and Liver Function

**FIGURE 5:**
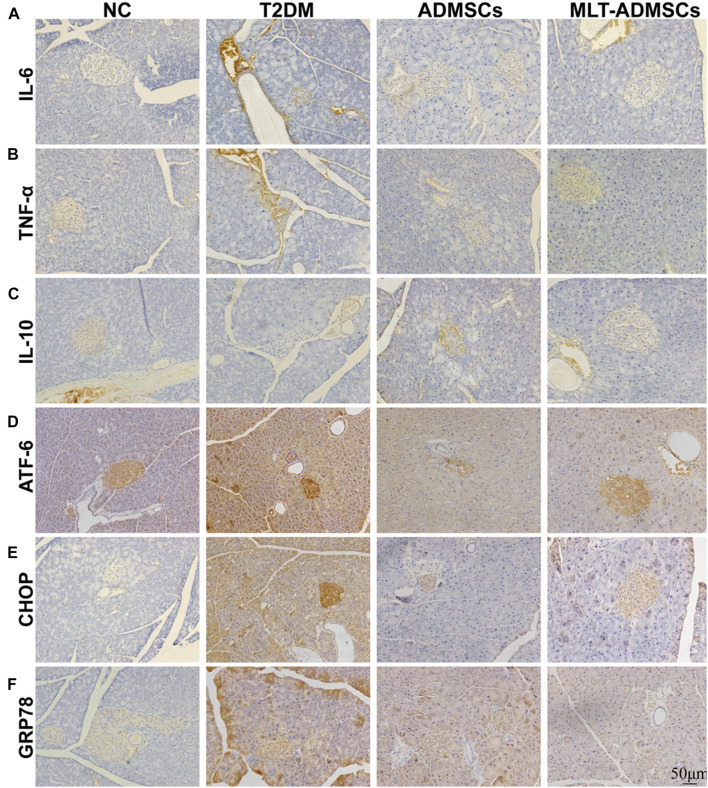
MLT promotes the therapeutic effect of ADMSCs through stronger anti-inflammatory and anti-ER stress abilities to restore pancreatic islet function. **(A)** Pancreatic IL-6 expression. **(B)** Pancreatic TNF-α expression. **(C)** Pancreatic IL-10 expression. **(D)** Pancreatic ATF-6 expression. **(E)** Pancreatic CHOP expression. **(F)** Pancreatic GRP78 expression.

Patients with T2DM often experience ER stress and chronic inflammation. The immunohistochemical staining showed that the ADMSCs alleviated islet inflammation and ER stress (Figures 5A–F). Inflammation and ER stress in islets can lead to injury to islet β cells. Restoring ER stress and inflammation in the islet microenvironment is beneficial for rebuilding islet β cells. ADMSCs promote the remodeling of pancreatic β-cells by repairing inflammation and ER stress levels in the islet microenvironment.

**FIGURE 6:**
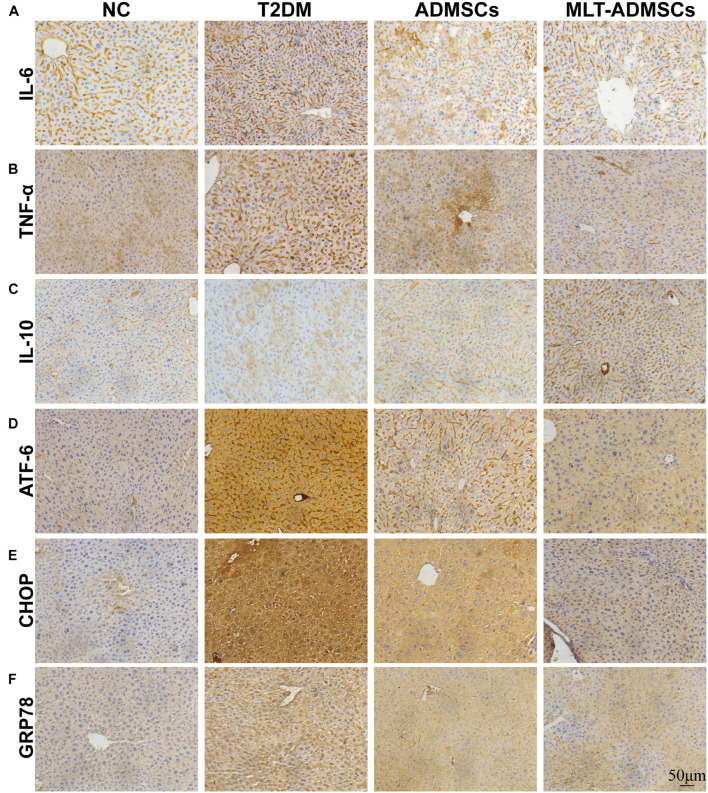
MLT promotes the therapeutic effect of ADMSCs through stronger anti-inflammatory and anti-ER abilities to restore liver function. **(A)** Liver IL-6 expression. **(B)** Liver TNF-α expression. **(C)** Liver IL-10 expression. **(D)** Liver ATF-6 expression. **(E)** Liver CHOP expression. **(F)** Liver GRP78 expression.

Type 2 diabetes mellitus causes liver disease and liver dysfunction. The mechanism involves the stimulation of liver glycogen metabolism and lipid metabolism disorders through hyperglycemia. Previous data have confirmed that ADMSCs alleviate liver damage caused by T2DM, but how they work remains unclear. The immunohistochemical staining showed that the therapeutic effect of ADMSCs in the liver was also related to the recovery of ER stress and inflammation (Figures 6A–F). In summary, the results show that the therapeutic effect of ADMSCs on T2DM is by restoring inflammation and ER stress. It is speculated that ADMSCs promote the recovery of multiple organs and have a therapeutic effect on T2DM from the perspective of the overall organ microenvironment.

### Melatonin Promotes the Therapeutic Effect of Adipose-Derived Mesenchymal Stem Cells in Canine DM

**FIGURE 7:**
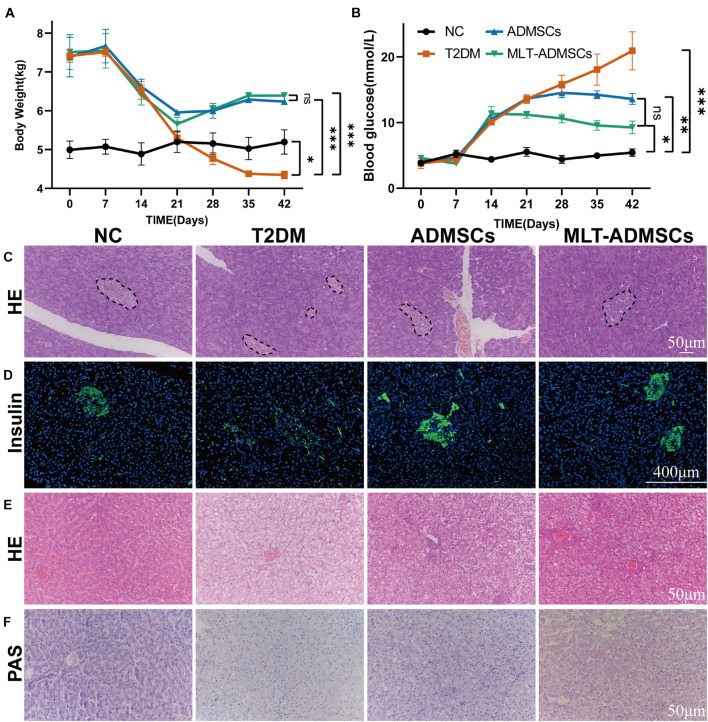
MLT promotes the therapeutic effect of ADMSCs in canine DM. **(A)** Weight changes. **(B)** Random blood glucose changes. **(C)** Islet H&E staining. **(D)** Insulin immunofluorescence staining. **(E)** Liver H&E staining. **(F)** Liver PAS staining. Values in this figure are the mean ± SE; *n* = 3 per group; ns > 0.05, ^∗^*p* < 0.05, ^∗∗^*p* < 0.01, ^∗∗∗^*p* < 0.001 determined by a repeated-measures ANOVA. The dotted ellipse includes the location and size of the islets.

The dog DM model was established by HFD and STZ. Seven days after the STZ injection, the dogs’ weight decreased, the blood glucose level increased, and the ADMSC treatment alleviated these symptoms (Figures 7A,B). In addition, H&E staining and insulin immunofluorescence staining were performed using each group of pancreases. The data show that MLT in dogs can also promote the recovery of hyperglycemia and insulin secretion by ADMSCs in dogs (Figures 7C,D). Similarly, the results of the liver H&E staining and PAS staining further confirmed the recovery effect of ADMSCs on liver damage and glucose metabolism in canine diabetes (Figures 7E,F). In dogs with DM, ADMSCs and MLT-ADMSCs have effects similar to those in mice.

### The Ability of Melatonin to Promote the Proliferation of Adipose-Derived Mesenchymal Stem Cells Depends on the Transforming Growth Factor β Pathway

**FIGURE 8:**
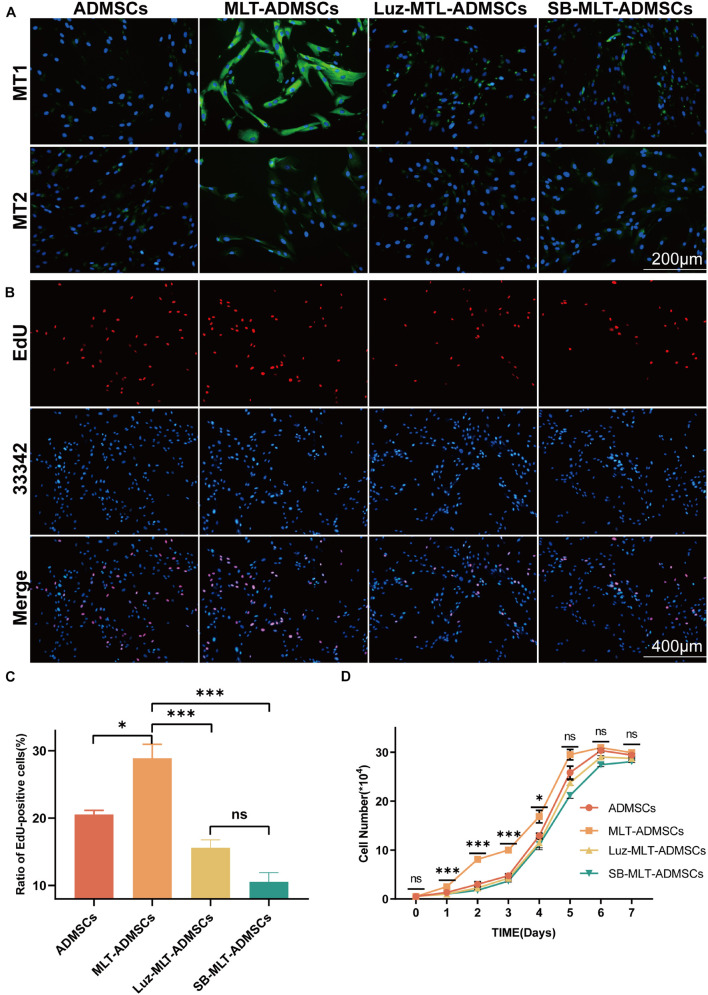
MLT depends on the MT1/MT2 receptor and activates the TGF-β pathway to promote the viability of ADMSCs. **(A)** MT1/MT2 immunofluorescence staining. **(B)** EdU staining. **(C)** EdU quantitative analysis. **(D)** Cell growth curve. Values in **(C,D)** are the mean ± SE; *n* = 3 per group; ns > 0.05, ^∗^*p* < 0.05, ^∗∗∗^*p* < 0.001 determined by a repeated-measures ANOVA.

**FIGURE 9:**
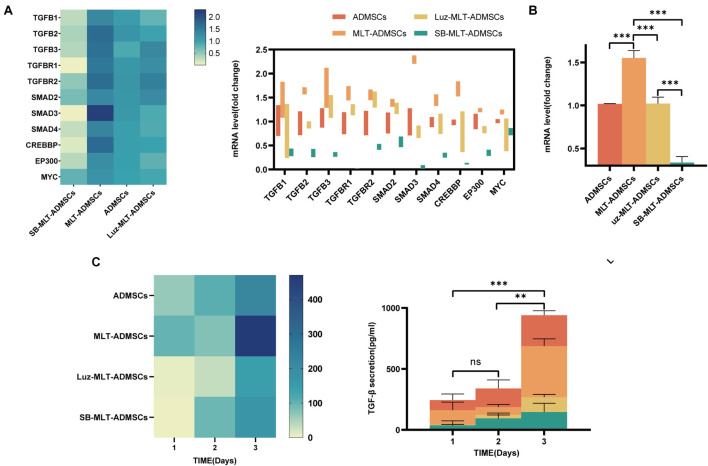
MLT activates the TGF-β pathway in ADMSCs. **(A)** TGFB expression level. **(B)** TGF-β pathway expression level. **(C)** ELISA was used to detect the amount of TGF-β secretion. Values in this figure are the mean ± SE; *n* = 3 per group; ns > 0.05, ^∗^*p* < 0.05, ^∗∗^*p* < 0.01, ^∗∗∗^*p* < 0.001 determined by a repeated-measures ANOVA.

Previous data have confirmed that MLT promotes the viability of ADMSCs *in vitro* (Figure 1), but the mechanism by which MLT regulates the viability of ADMSCs is unknown. The MLT receptor inhibitor luzindole and the TGF-βR1/ALK5 inhibitor SB431542 were combined with MLT to treat ADMSCs. When luzindole was used to inhibit MT1/MT2, the expression of MT1/MT2 in the ADMSCs was inhibited (Figure 8A), and cell proliferation slowed (Figures 8B–D). This finding explains why the effect of MLT on the activity of ADMSCs depends on its combination with MT1/MT2. The TGF-β pathway includes TGF-β ligands (TGF-β1, TGF-β2, and TGF-β3), TGF-β receptors (TGF-βR1 and TGF-βR2) and downstream Smad and Smad-related transcription factors. When the TGF-β inhibitor SB431542 was added, the cell proliferation caused by MLT was inhibited (Figures 8B–D). Inhibited TGF-β receptor, MLT still binds to MT1/MT2 (Figure 8A). The above results indicated that MLT firstly binds to the MT1/MT2 of ADMSCs, thereby affecting the TGF-β pathway. The expression of genes related to the TGF-β pathway was detected; it was found that MLT activated the TGF-β pathway (Figure 9A). With the activation of the cell cycle-related genes MYC/CREBBP/EP300, it is speculated that MLT activates the TGF-β pathway to promote the cell cycle renewal of ADMSCs, thereby promoting the viability of ADMSCs (Figure 9A). In addition, when the binding of MLT to MT1/MT2 was inhibited, the overall expression of TGF-β family and downstream genes was decreased (Figure 9B). The above results proved that MLT first combines with MT1/MT2 and then activates the TGF-β family, thereby affecting the cell cycle and promoting cell viability. Surprisingly, MLT promoted the ADMSCs to secrete more TGF-β, which explains the increased expression of the TGF-β receptors TGF-βR1 and TGF-βR2 and may be related to the mechanism by which MLT promotes the efficacy of ADMSCs (Figure 9C).

### Inhibition of the Transforming Growth Factor β Pathway Blocked the Promotion of Adipose-Derived Mesenchymal Stem Cell Efficacy by Melatonin

**FIGURE 10:**
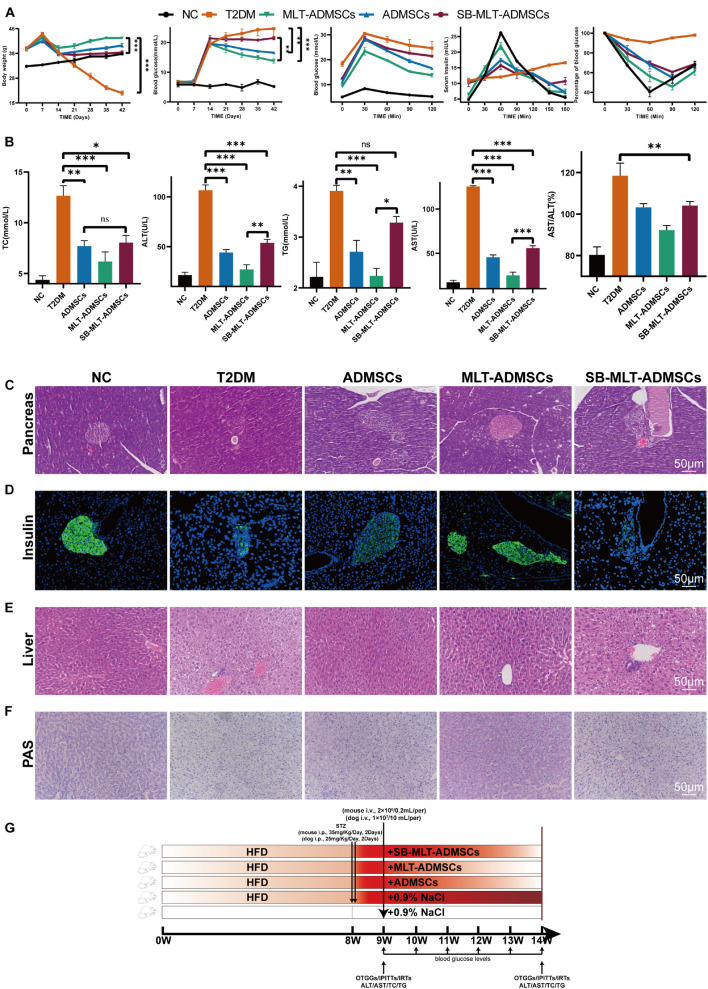
Inhibition of the TGF-β pathway blocked the promotion of the efficacy of ADMSCs by MLT. **(A)** Levels of blood glucose metabolism and insulin resistance, including random blood glucose levels, OTGG, IRTs, and IPITT. **(B)** Liver function and glucose metabolism, including TC, TG, ALT, AST, and AST/ALT. **(C)** Pancreas H&E staining. **(D)** Pancreas immunofluorescence of insulin. **(E)** Liver H&E staining. **(F)** Liver PAS staining. **(G)** Experimental design. Values in this figure are the mean ± SE; *n* = 5 mice per group; ns > 0.05, ^∗^*p* < 0.05, ^∗∗^*p* < 0.01, ^∗∗∗^*p* < 0.001 determined by a repeated-measures ANOVA.

To determine the function of TGF-β on the therapeutic effect of ADMSCs, MLT-ADMSCs treated with SB431542 were used to treat T2DM mice (Figure 10G). When TGF-β is inhibited, the recovery effect of ADMSCs on blood sugar levels, insulin resistance levels and islet remodeling is weakened (Figures 10A,C,D and Supplementary Figure 2). When TGF-β is inhibited, the recovery of liver function is weakened (Figures 10B,E). In addition, inhibiting TGF-β reduced the recovery effect on liver glycogen metabolism (Figure 10F). In summary, TGF-β is the key factor by which MLT impacts ADMSCs.

### Transplantation of Adipose-Derived Mesenchymal Stem Cells and Melatonin-Adipose-Derived Mesenchymal Stem Cells Is Safe for Animals

**FIGURE 11:**
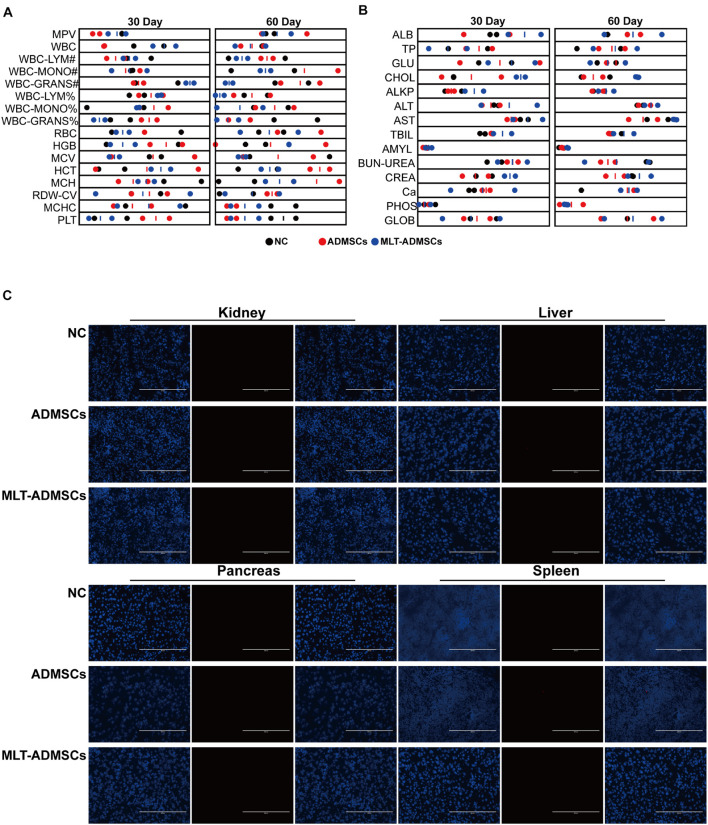
Transplantation of ADMSCs and MLT-ADMSCs is safe for animals. **(A)** Routine blood examination. **(B)** Biochemical analysis. **(C)** PKH26 staining.

To determine the safety of ADMSCs and MLT-ADMSCs, healthy dogs were injected with ADMSCs and MLT-ADMSCs. The dogs’ physiological data were regularly checked, and residual ADMSCs were detected after 60 days. The physiological indicators of all dogs were within the normal range (Figures 11A,B). After 60 days, the residual status of ADMSCs was detected. ADMSCs and MLT-ADMSCs were not detected in the liver, kidney, pancreas, or spleen (Figure 11C). In general, all MSCs were cleared after 60 days, and there were no side effects in the dogs.

## Discussion

Thus far, many studies investigated the effect of MSCs treatment on various diseases. The main point of view is that MSCs differentiate into insulin-secreting cells, promote the regeneration of pancreatic islet β cells, protect endogenous pancreatic β cells, restore insulin resistance, improve glucose metabolism, etc., to achieve therapeutic effects (Rodríguez-Lozano et al., 2015; Zang et al., 2017; Hu and Li, 2019; Li et al., 2021; Xue et al., 2021). Studies have shown that MSC treat diseases by restoring body inflammation, ER stress, oxidative stress, autophagy, etc (Volarevic et al., 2011; Davey et al., 2014; Zang et al., 2017; Yu et al., 2019; He et al., 2020; Elshemy et al., 2021). However, there are still many problems in treatment using MSCs. Various unfavorable factors *in vivo* and *in vitro* affect the cell state of MSCs and hinder their therapeutic effect (Farahzadi et al., 2018; Hu and Li, 2019). Long-term *in vitro* culture leads to reduced MSC proliferation ability, senescence, and morphological changes (Danisovic et al., 2017; Farahzadi et al., 2018). Furthermore, 80–90% of ADMSCs died within 72 h after transplantation (Liu et al., 2009; Jeong and Cho, 2016; Danisovic et al., 2017). The low survival rate and proliferation rate of MSCs after transplantation are mainly due to the lack of nutrients or growth factors needed by MSCs in the body. Furthermore, adverse factors, such as oxidative stress and chronic inflammation in the body, affect the survival of MSCs *in vivo* (Han et al., 2016). Thus far, studies have attempted to promote the viability of MSCs *in vivo* and *in vitro*, but the effect is still not satisfactory, and research aiming to promote cell viability is lacking (Liu et al., 2009; Chen et al., 2014; Lee et al., 2014; Tang et al., 2014; Han et al., 2016; Shuai et al., 2016; Kadry et al., 2018).

Melatonin plays an important physiological role in the human body, and it has been determined that MLT plays an important role in the regulation of the circadian rhythm (Redman et al., 1983; McArthur et al., 1997). There are many advantages to using MLT for MSC culture. MLT are used as a component of cytoprotective agents to protect MSCs from oxidative stress, inflammation, apoptosis, and aging to regulate the cellular state of MSCs *in vivo* and *in vitro* (Calvo et al., 2013; Ma et al., 2013). Studies have shown that MLT promotes the proliferation and osteogenic differentiation of MSCs *in vitro* (Luchetti et al., 2010, 2014; Rodríguez-Lozano et al., 2015). In this study, we used MLT as an additive in the culture of ADMSCs *in vitro*. The results indicate that MLT promotes the viability of ADMSCs cultured *in vitro*. To explore how MLT affects ADMSCs, we used luzindole combined with MLT to treat ADMSCs. When the two receptors MT1/MT2 of MLT are inhibited, the pro-proliferation effect of MLT on ADMSCs disappears.

The TGF-β family is an important cytokine in the body that regulates cell proliferation and differentiation (Blobe et al., 2000). Studies have noted that TGF-β is essential for the proliferation of ADMSCs (Ng et al., 2008). We tested the expression of the TGF-β family and its downstream Smad and cell cycle-related transcription factors. After the MLT treatment, the ADMSCs expressed more TGF-β; the expression levels of TGF-βR1, TGF-βR2 and downstream Smad and cell cycle-related transcription factors also increased. We speculate that the proliferation of ADMSCs by MLT may be related to TGF-β. Then, we inhibited the TGF-βR1 of ADMSCs. It was found that when TGF-β was inhibited, the promotion of MLT on ADMSCs disappeared. Interestingly, the inhibition of TGF-β does not affect the expression of MT1/MT2. We also detected TGF-β in the culture medium of ADMSCs after MLT treatment. The above results explain the mechanism by which MLT promotes the activity of ADMSCs. The combination of MLT and MT1/MT2 promotes the secretion of TGF-β from ADMSCs. MLT improves the cell viability of ADMSCs through the TGF-β pathway.

Subsequently, we used ADMSCs pretreated with MLT to treat T2DM mice and dogs. We found that ADMSCs ameliorated the islet damage and liver damage caused by T2DM. The pancreas and liver are the key organs in T2DM, and their damage leads to T2DM. Two characteristics of T2DM are hyperglycemia and insulin resistance. Damage to pancreatic β cells leads to a decrease in insulin secretion, while liver damage leads to a decrease in the sensitivity of the liver to insulin, leading to hyperglycemia and insulin resistance. The underlying mechanism is mainly due to impaired liver glucose metabolism caused by hyperglycemia stimulation, resulting in insulin resistance, which, in turn, leads to pancreatic β cell damage (American Diabetes Association, 2015).

To determine whether TGF-β is a key factor in MLT function, we used MLT-ADMSCs that inhibit TGF-β to treat T2DM. The results showed the key role of TGF-β. After inhibiting the secretion of TGF-β, the effect of MLT almost disappeared and was even weaker than that of ADMSCs. This result reveals the key role of TGF-β in MLT function. Moreover, the inhibition of TGF-β reduces the efficacy of ADMSCs to a lower level, implying that TGF-β plays a key role in the treatment of MSCs.

Some studies report that ER stress and chronic inflammation are also potential killers of T2DM and cause other complications of T2DM (Hu et al., 2018). Inflammation and ER stress are self-protection measures caused by the body to resist unfavorable factors, but their abnormal occurrence is harmful to the body. We detected the inflammation and ER stress levels in T2DM mice. When T2DM occurs, inflammation and ER stress coexist. After the ADMSC treatment, liver and pancreas inflammation and ER stress were improved. The MLT pretreatment of ADMSCs strengthens these effects. We detected more TGF-β in the culture medium of the ADMSCs after the MLT treatment. The promoting effect of MLT on the efficacy of ADMSCs includes two aspects. First, MLT promotes cell viability and achieves a better therapeutic effect in the body. Second, MLT promotes ADMSCs to secrete more TGF-β, which may be related to a stronger anti-inflammatory and anti-ER stress. In addition, existing studies mostly use human-derived MSCs to treat mouse T2DM models, and xenogeneic therapy has many defects. We used canine-derived ADMSCs to treat mouse and canine disease models, which simulated xenogeneic therapy and allogeneic therapy well, provided a new solution for clinical research concerning MSC therapy and solved the ethical limitations of MSC homologous therapy. Furthermore, as a companion animal of humans, dogs have a living environment and habits that are closer to those of humans. Dogs are a good animal disease model and serve as good subjects for the clinical advancement of MSC treatment.

Melatonin promotes the differentiation ability of MSCs. Off-target differentiation and cancer cell transformation have always plagued the treatment of MSCs. In previous studies, the effect of MLT on the differentiation ability of ADMSCs was partially identified, proving the salvage effect of MLT on the osteogenic differentiation potential of aging ADMSCs. In this study, we observed the cell morphology of ADMSCs treated with MLT, performed morphological staining (Figures 1A,B), and confirmed that the morphology of MSCs was normal. We transplanted ADMSCs and MLT-ADMSCs into healthy dogs, and the results showed that ADMSCs and MLT-ADMSCs are safe. There was no residual phenomenon 60 days after the treatment.

Unfortunately, this experiment did not further explore whether TGF-β is directly related to the efficacy of MSCs. More research concerning the function of TGF-β in the treatment of MSCs is needed in the future. There is still a lack of stronger evidence exploring more mechanisms by which MLT promotes the efficacy of ADMSCs. Our research did not prove the relationship between ADMSCs’ anti-inflammatory effect and anti-endoplasmic reticulum stress. We know that ADMSCs were anti-inflammatory and anti-ER stress in the body. Since ER stress and inflammation often occur at the same time, it is urgent to research the relationship between endoplasmic reticulum stress and inflammation. Furthermore, as a new animal disease model, more research is needed to establish canine disease models that mimic human diseases. It is necessary to further explore the treatment mechanism of ADMSCs to support the use of ADMSCs in clinical treatment.

## Conclusion

In this study, MLT was used as an additive to culture ADMSCs *in vitro*. MLT binds MT1/MT2 and activates the TGF-β pathway, thereby affecting the cell cycle changes of ADMSCs and promoting the viability of ADMSCs. ADMSCs primed with MLT were used to treat T2DM in mice and dogs. ADMSCs restore hyperglycemia, insulin resistance, insulin sensitivity, and glucose metabolism in T2DM by restoring inflammation and ER stress in the pancreas and liver. The MLT improved the anti-inflammatory and anti-ER stress abilities of ADMSCs through TGF-β and improved the therapeutic effect, and which is safe and valuable for pet clinic.

## Data Availability Statement

The original contributions presented in the study are included in the article/Supplementary Material, further inquiries can be directed to the corresponding author.

## Ethics Statement

The animal study was reviewed and approved by the Ethics Committee of Northwest A&F University for the Use of Laboratory Animals.

## Author Contributions

BL and JH conceived and designed the study. BL, XC, WH, and AA performed the animal experiments. BL, XC, and AA carried out the cell experiments. BL, MZ, NT, and ZK performed the molecular biology experiments. JD, BL, WJ, SP, and HT analyzed the data. BL drafted the manuscript. JH, BL, and JD revised and edited the manuscript. All authors have read and approved the final version of the manuscript.

## Conflict of Interest

The authors declare that the research was conducted in the absence of any commercial or financial relationships that could be construed as a potential conflict of interest.

## Publisher’s Note

All claims expressed in this article are solely those of the authors and do not necessarily represent those of their affiliated organizations, or those of the publisher, the editors and the reviewers. Any product that may be evaluated in this article, or claim that may be made by its manufacturer, is not guaranteed or endorsed by the publisher.
