# Corrigendum: Melatonin Promotes the Therapeutic Effect of Mesenchymal Stem Cells on Type 2 Diabetes Mellitus by Regulating TGF-β Pathway

**DOI:** 10.3389/fcell.2021.799571

**Published:** 2021-12-07

**Authors:** Balun Li, Xuedi Cheng, Aili Aierken, Jiaxin Du, Wenlai He, Mengfei Zhang, Ning Tan, Zheng Kou, Sha Peng, Wenwen Jia, Haiyang Tang, Jinlian Hua

**Affiliations:** ^1^ Shaanxi Centre of Stem Cells Engineering and Technology, College of Veterinary Medicine, Northwest A&F University, Xianyang, China; ^2^ Department of Animal Engineering, Yangling Vocational and Technical College, Xianyang, China; ^3^ Department of Veterinary Medicine, College of Animal Sciences, Institute of Preventive Veterinary Sciences, Zhejiang University, Hangzhou, China; ^4^ Shanghai East Hospital, East Hospital Affiliated to Tongji University, Shanghai, China; ^5^ State Key Laboratory of Respiratory Disease, Guangzhou Institute of Respiratory Health, The First Affiliated Hospital of Guangzhou Medical University, Guangzhou, China

**Keywords:** melatonin, adipose-derived mesenchymal stem cells, type 2 diabetes mellitus, TGF-β, inflammation, canine

In the published article, there was an error regarding the affiliation for Haiyang Tang. As well as having affiliation 1, they should also have “*State Key Laboratory of Respiratory Disease, Guangzhou Institute of Respiratory Health, The First Affiliated Hospital of Guangzhou Medical University, Guangzhou, China*.”

The authors apologize for this error and state that this does not change the scientific conclusions of the article in any way. The original article has been updated.

