# Supplementary material for: Melatonin Promotes the Therapeutic Effect of Mesenchymal Stem Cells on Type 2 Diabetes Mellitus by Regulating TGF-β Pathway
Source: Front Cell Dev Biol. 2021 Oct 15;9:722365. doi: 10.3389/fcell.2021.722365 (PMC8554153; doi:10.3389/fcell.2021.722365)
Supplement: Supplementary file 1 [file Data_Sheet_1.docx]

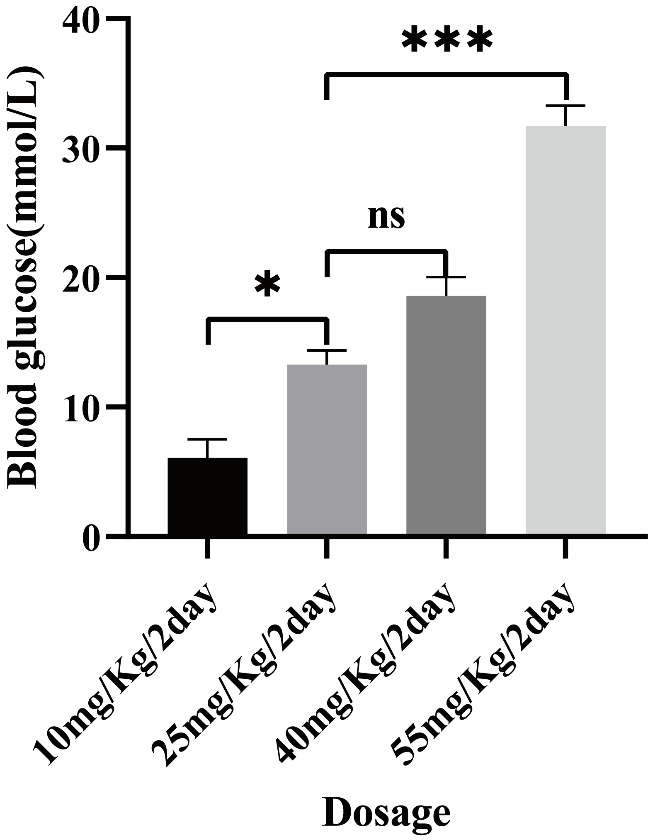


Supplement figure 1. Different doses of STZ transplantation to treat canine T2DM model.


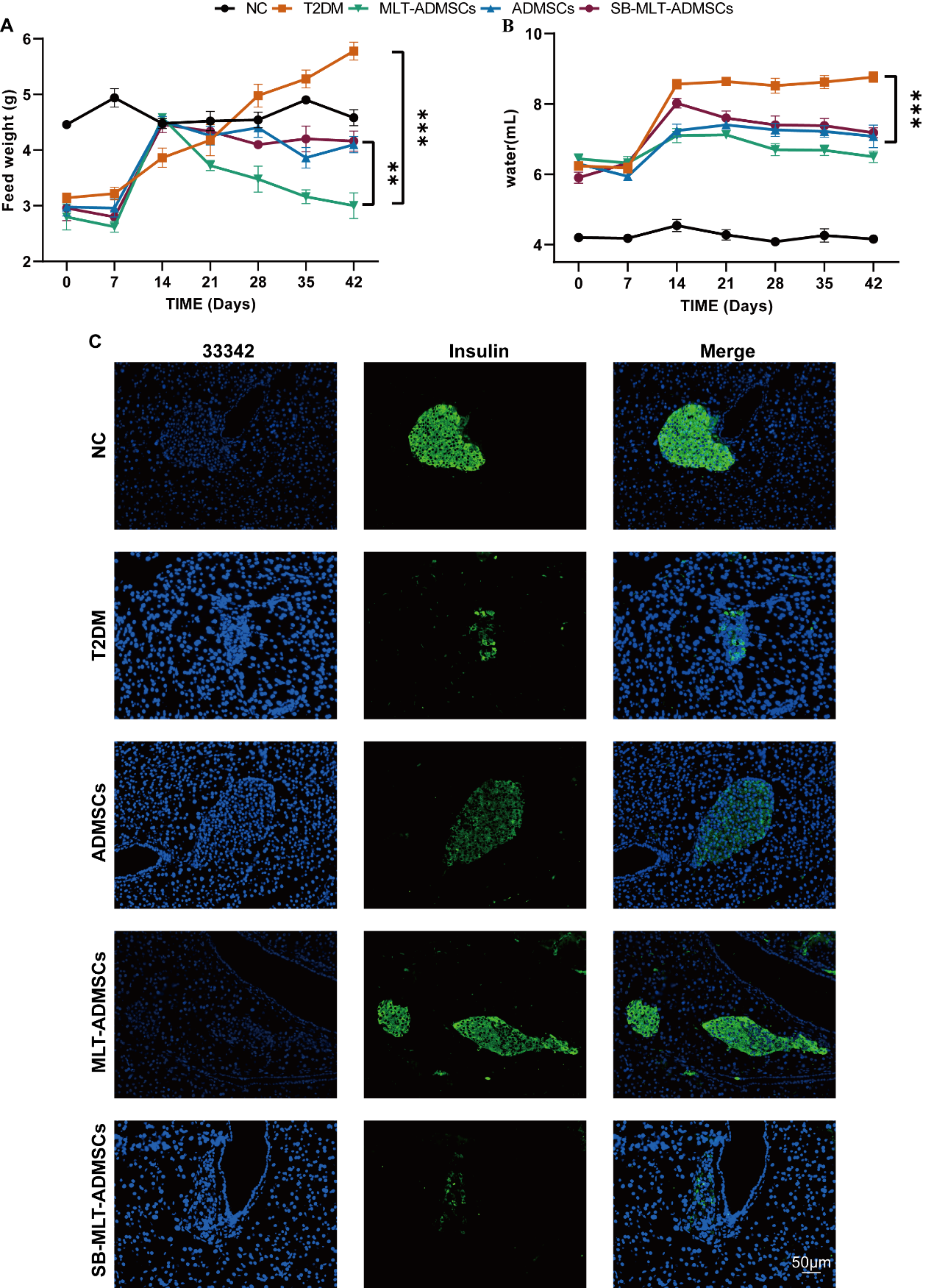


Supplement figure 2. Inhibition of the TGF-β pathway blocks the effects of MLT

Supplementary table1.Sequences of real-time PCR primers

| mRNA | Primer |  | Sequences(5'--3') | Annealing temperature(℃) | Fragment size(bp) |
| --- | --- | --- | --- | --- | --- |
| Dog-GAPDH | Forward primer |  | GCTGCCAAATATGACGACATCA | 59.35 | 75 |
|  | Reverse primer |  | GTAGCCCAGGATGCCTTTGAG | 60.79 |  |
| Dog-TGFB1 | Forward primer |  | TGACCTTTCTGCCTTCGACC | 59.97 | 295 |
|  | Reverse primer |  | TCCTAGTGCCCTACAGTCCC | 60.03 |  |
| Dog-TGFB2 | Forward primer |  | GAAGACTACCCCGAACCCGA | 60.97 | 172 |
|  | Reverse primer |  | AGAAGGGCGGCATGTCTATC | 59.61 |  |
| Dog-TGFB3 | Forward primer |  | TGAGGACACATTGAAGCGGA | 59.32 | 88 |
|  | Reverse primer |  | CCTCTCCTCGCTTGCTTGAC | 60.74 |  |
| Dog-TGFBR1 | Forward primer |  | AGGCTTACAGCTTTGCGGAT | 60.04 | 245 |
|  | Reverse primer |  | CAGCTGTTTCCTGGGTCCAA | 60.18 |  |
| Dog-TGFBR2 | Forward primer |  | TATGCCTCCTGGAAGACGGA | 60.03 | 172 |
|  | Reverse primer |  | CATGCCGAGTGAGGTACTCC | 59.9 |  |
| Dog-SMAD2 | Forward primer |  | CACTCCGCCTGTTGTGAAGA | 60.25 | 184 |
|  | Reverse primer |  | TTGAGTGGTGATGGCTTTCTCA | 59.89 |  |
| Dog-SMAD3 | Forward primer |  | TCCTGGCTCAATCCGTCAAC | 60.04 | 224 |
|  | Reverse primer |  | AAACACACTGGAACAGCGGA | 60.11 |  |
| Dog-SMAD4 | Forward primer |  | CCCATCCCGGACATTACTGG | 59.89 | 89 |
|  | Reverse primer |  | AGGTAAACAGGAGAGAACATCAGG | 59.78 |  |
| Dog-CREBBP | Forward primer |  | TGAACCCCCAGTTATCCAGC | 59.38 | 253 |
|  | Reverse primer |  | ACCTCTCCGTTCGCTTGTTC | 60.32 |  |
| Dog-EP300 | Forward primer |  | TGCTGTGGCAGAAAGTTGGA | 60.11 | 174 |
|  | Reverse primer |  | GGAAGGGTCATCCCCCAAAG | 60.03 |  |
| Dog-MYC | Forward primer |  | AGGAACGAGCTGAAACGGAG | 60.04 | 84 |
|  | Reverse primer |  | CACTACCTTGGGGGCCTTTT | 59.89 |  |
